# Transcriptional Profiling of Mouse Eosinophils Identifies Distinct Gene Signatures Following Cellular Activation

**DOI:** 10.3389/fimmu.2021.802839

**Published:** 2021-12-14

**Authors:** Avishay Dolitzky, Guy Shapira, Sharon Grisaru-Tal, Inbal Hazut, Shmulik Avlas, Yaara Gordon, Micahl Itan, Noam Shomron, Ariel Munitz

**Affiliations:** ^1^ Department of Clinical Microbiology and Immunology, Faculty of Medicine, Tel Aviv University, Tel Aviv, Israel; ^2^ Department of Cell and Developmental Biology, Faculty of Medicine, Tel Aviv University, Tel Aviv, Israel

**Keywords:** eosinophils, heterogeneity, activation, IL-4, inflammation, IFN-γ

## Abstract

Eosinophils are multifunctional, evolutionary conserved leukocytes that are involved in a plethora of responses ranging from regulation of tissue homeostasis to host defense and cancer. Eosinophils have been studied mostly in the context of Type 2 inflammatory responses such as those found in allergy. Nonetheless, it is now evident that they participate in Type 1 inflammatory responses and can respond to Type 1 cytokines such as IFN-γ. Recent data suggest that the pleotropic roles of eosinophils are due to heterogeneous responses to environmental cues. Despite this, the activation profile of eosinophils, in response to various stimuli is yet to be defined. To better understand the transcriptional spectrum of eosinophil activation, we exposed eosinophils to Type 1 (e.g. IFN-γ, *E. coli*) vs. Type 2 (e.g. IL-4) conditions and subjected them to global RNA sequencing. Our analyses show that IL-4, IFN-γ, *E. coli* and IFN-γ in the presence of *E. coli* (IFN-γ/*E. coli*)-stimulated eosinophils acquire distinct transcriptional profiles, which polarize them towards what we termed Type 1 and Type 2 eosinophils. Bioinformatics analyses using Gene Ontology based on biological processes revealed that different stimuli induced distinct pathways in eosinophils. These pathways were confirmed using functional assays by assessing cytokine/chemokine release (i.e. CXCL9, CCL24, TNF-α and IL-6) from eosinophils following activation. In addition, analysis of cell surface markers highlighted CD101 and CD274 as potential cell surface markers that distinguish between Type 1 and Type 2 eosinophils, respectively. Finally, the transcriptome signature of Type 1 eosinophils resembled that of eosinophils that were obtained from mice with experimental colitis whereas the transcriptome signature of Type 2 eosinophils resembled that of eosinophils from experimental asthma. Our data demonstrate that eosinophils are polarized to distinct “Type 1” and “Type 2” phenotypes following distinct stimulations. These findings provide fundamental knowledge regarding the heterogeneity of eosinophils and support the presence of transcriptional differences between Type 1 and Type 2 cells that are likely reflected by their pleotropic activities in diverse disease settings.

## Introduction

Eosinophils are evolutionary conserved bone marrow-derived granulocytes. Under baseline conditions they reside mainly in tissues such as the gastrointestinal (GI) tract, spleen, adipose tissue, lymph nodes and thymus, where they have important regulatory and homeostatic roles (1, 2). During Type 2-associated immune responses such as those found in allergic diseases and parasitic infections, eosinophils are recruited to the tissue and their accumulation is a hallmark disease characteristic and therapeutic target (1, 3). Nonetheless, accumulating evidence suggest that eosinophils have important roles in Type 1 immune responses as well. For example, eosinophil extracellular traps were shown to participate in mucosal host defense immunity towards bacteria (4). Furthermore, eosinophils display anti-viral activity in response to respiratory syncytial virus and parainfluenza (5, 6). They are also associated with several chronic Type 1-associated inflammatory diseases such as inflammatory bowel disease and rheumatoid arthritis (7, 8). Collectively, these data suggest that eosinophils are capable of responding to distinct environmental cues that are likely dictated by the nature of the inflammatory response and the anatomical location where it occurs.

Eosinophils express multiple cell surface receptors including receptors for cytokines, chemokines, immunoglobulins (Ig), complement component and Ig-superfamily receptors (9). Engagement of such receptors on eosinophils can lead to their degranulation and secretion of highly specific cationic granule proteins. In addition, it may result in the generation and secretion of a wide range of cytokines, lipid-derived mediators and neuro-mediators (9). Although much is known regarding the repertoire of eosinophil cell surface receptor expression, the transcriptional landscape of eosinophil responses to distinct stimuli is still unclear. This is of specific interest since until recently, eosinophils were considered terminally differentiated cells with little to no heterogeneity. Nonetheless, recent data challenged this paradigm and suggested that eosinophils, like additional myeloid cells, could be polarized into different activation states and display marked phenotypic heterogeneity (10). For example, CD16^hi^ eosinophils were capable of suppressing T cell activities more potently than CD16^low^ eosinophils (11). Furthermore, differential expression of eosinophil surface markers suggested the presence of several eosinophil populations distinguished by different levels of Siglec-F in the bone marrow, spleen, and peripheral blood. Moreover, two distinct eosinophil subsets were characterized in the lungs of mice following allergen challenge (12). These subsets differed in their transcriptional profile, surface phenotype (Siglec-F^int^/CD62^+^/CD101^low^ *vs.* Siglec-F^hi^/CD62^-^/CD101^hi^) anatomical location (parenchymal vs. peribronchial), nuclear morphology (ring-shaped vs. segmented), and dependence on IL-5, as well as their ability to down-modulate Type 2 immune responses (12). In contrast, we have recently shown that in the lungs of mice with experimental breast cancer metastasis, these eosinophil populations display no transcriptional difference (13). Thus, it is currently unknown whether these markers represent distinct eosinophil subtypes or rather a continuum of eosinophil activation states.

It has been established that myeloid cells such as macrophages, dendritic cells and neutrophils display functional heterogeneity in response to different environmental triggers (14, 15). Exposure of these cells to Type 1-associated cytokines (e.g. IFN-γ in the presence of bacterial stimuli) polarized them to display a pro-inflammatory phenotype (often termed M1 or N1). In contrast, exposure to Type 2-associated cytokines (e.g. IL-4 or IL-13) drove them towards immunomodulatory activities (often termed M2 or N2) (14). Whether IFN-γ and IL-4 instruct distinct transcriptional activity in eosinophils remains to be defined. Furthermore, the similarity between *in vitro* polarized eosinophils and macrophages, or between them and eosinophils from different disease states that may represent Type 1 or Type 2 inflammatory environments is unknown.

Herein, we aimed to characterized the transcriptional profile of mouse eosinophils in response to stimuli that are associated with Type 1 and Type 2 environments. We demonstrate that IL-4-stimulated eosinophils were markedly different than eosinophils that were activated with IFN-γ or IFN-γ + *E. coli*. We further show that IL-4-activated eosinophils shared a transcriptome signature that was similar to eosinophils from Type 2-associated diseases (e.g. asthma) whereas IFN-γ + *E. coli-*activated eosinophils were similar to eosinophils from a Type 1 associated disease (e.g. colitis). Finally, we suggest CD101 and CD274 as cell surface markers that can distinguish between Type 1 and Type 2-associated eosinophils, respectively. Our results contribute to the growing understanding regarding the heterogeneity of eosinophils and describe important markers of their polarization.

## Materials and Methods

### Mice

C57BL/6 WT and C57BL/6 NJ.1638 *Il5^Tg^* mice (Kindly provided by Dr. James L. Lee, Mayo Clinic, Phoenix, USA) were used for all studies using primary mouse cells. The mice were housed under specific pathogen-free conditions. All experiments were reviewed and approved by the Animal Health Care Committee of the Tel Aviv University and were performed in accordance with the regulations and guidelines regarding the care and use of animals for experimental procedures.

### Eosinophil Isolation

Mouse eosinophils were isolated from the peritoneal cavity of *Il5^Tg^* mice under sterile conditions. Peritoneal cavity was washed with 10mL of PBS. Thereafter, negative selection of eosinophils was performed using anti-Ty1.2 (11443D, Invitrogen) and anti-B220 (11331D, Invitrogen) Dynabead-conjugated antibodies according to the manufacturer’s instructions. Eosinophil purity was validated using flow cytometry; Eosinophils were used when purity >95% and viability > 95%.

### Stimulation of Eosinophils

Eosinophils were incubated with DMEM supplemented with 10% fetal calf serum and stimulated with IL-4 (100 ng/ml),10^-3^ heat inactivated *E. coli*, INF-γ (50 ng/ml), or combination of INF-γ and heat inactivated *E. coli* (50 ng/ml and 10^-3^). Stimulated eosinophils were than incubated at 37°C for 18hrs.

### RNA Sequencing

RNA was extracted using TRIzol™ Reagent (Invitrogen) according to the manufacturer’s instructions. The RNA integrity number (RIN) was analyzed using Typestation (Agilent) and only samples of RIN>8 were used. RNA samples were prepared using the CEL-Seq2 protocol (16) with minor changes: instead of single-cells as input, 2 ng of purified RNA was used for library preparation. The CEL-Seq library was run on an Illumina NextSeq 550 apparatus according to manufacturer’s recommendation. The number of reads ranged from 3,093,819 to 10,621,072 per sample. The reads were mapped to the Mus musculus, GRCm38 genome (fasta:ftp://ftp.ensembl.org/pub/release97/fasta/mus_musculus/pep/Mus_musculus.GRCm38.pep.abinitio.fa.gzgtfftp://ftp.ensembl.org/pub/release97/gtf/mus_musculus/Mus_musculus.GRCm38.97.chr_patch_hapl_scaff.gtf.gz) using Tophat2 version 2.1.0 (17), with up to 2 mismatches allowed per read, the minimum and maximum intron sizes were set to 50 and 100,000, respectively, and an annotation file was provided to the mapper. The percentage of uniquely mapped reads ranged from 2,599,806 to 8,909,751 per sample. Only uniquely mapped reads were counted to genes, using ‘HTSeq-count’ package version 0.6.1 with ‘union’ mode (18). Normalization and differential expression analyses were conducted using DESeq2 R package version 1.10.0 (19). Sample preparation, sequencing, quality control, and normalization were conducted by the Technion Genome Center, Life Science and Engineering Interdisciplinary Research Center, Technion, Haifa, Israel.

### Bioinformatics Analysis

The number of reads ranged from 3,093,819 to 10,621,072 per sample. The reads were mapped to the Mus musculus, GRCm38 genome using Tophat2 version 2.1.0 (17) with up to 2 mismatches allowed per read, the minimum and maximum intron sizes were set to 50 and 100,000, respectively. The percentage of uniquely mapped reads ranged from 2,599,806 to 8,909,751 per sample. Only uniquely mapped reads were counted to genes, using ‘HTSeq- count’ package version 0.6.1 with ‘union’ mode (18) RNA-Seq data from the experiment was trimmed using fastp 0.20.1 (20) and aligned using STAR 2.7.2a (21). Normalization and differential expression analyses were conducted using DESeq2 R package version 1.32.0 (19). Genes were regarded as statistically significantly and differentially expressed if they presented false discovery rate (FDR) lower than 0.05, and changed their expression by a factor of two or more. P values were adjusted with FDR multiple comparison correction (22). Gene ontology annotations were obtained from Ensembl and pathway graphs were obtained from KEGG. In several analyses, datasets were retrieved from public domains and therefore not all genes were identified. In such cases *NA* represents non-applicable. The datasets presented in this study can be found online in accession number GSE189213.

### Enzyme-Linked Immunosorbent Assay (ELISA)

Cytokine levels were measured by commercially available enzyme-linked immunosorbent assay (ELISA) kit according to manufacturer instructions: CXCL9, IL-6, TNF-α, and CCL-17 (R&D Systems, Minneapolis, MN).

### Flow Cytometry

Flow cytometry was performed using a Gallios flow cytometer (Beckman-Coulter). To validate the expression level of selected surface markers, isolated eosinophils (3 X 10^5^ cells in 200 μl) were activated with IL-4 (100ng/ml) or INF-γ (50ng/ml) and incubated at 37°C for 18 hours. Thereafter, the expression of CD274 or CD101 were determined by staining with anti-mouse CD274-PE (Biolegend) and anti-CD101-APC (Milteny) antibodies or isotype control (Rat IgG2b, Biolegend). Stainings were performed on ice for 30 minutes in HBSS supplemented with 1% BSA, 0.1% sodium azide. Data were analyzed using Kaluza analysis software on 10,000-50,000 acquired events. Surface molecule expression was calculated by defining the delta mean fluorescent intensity between the specific antibody stain and the isotype-matched control antibody.

## Results

### Differential Activation of Eosinophils Results in Induction of Unique Transcriptional Programs

**Figure 1:**
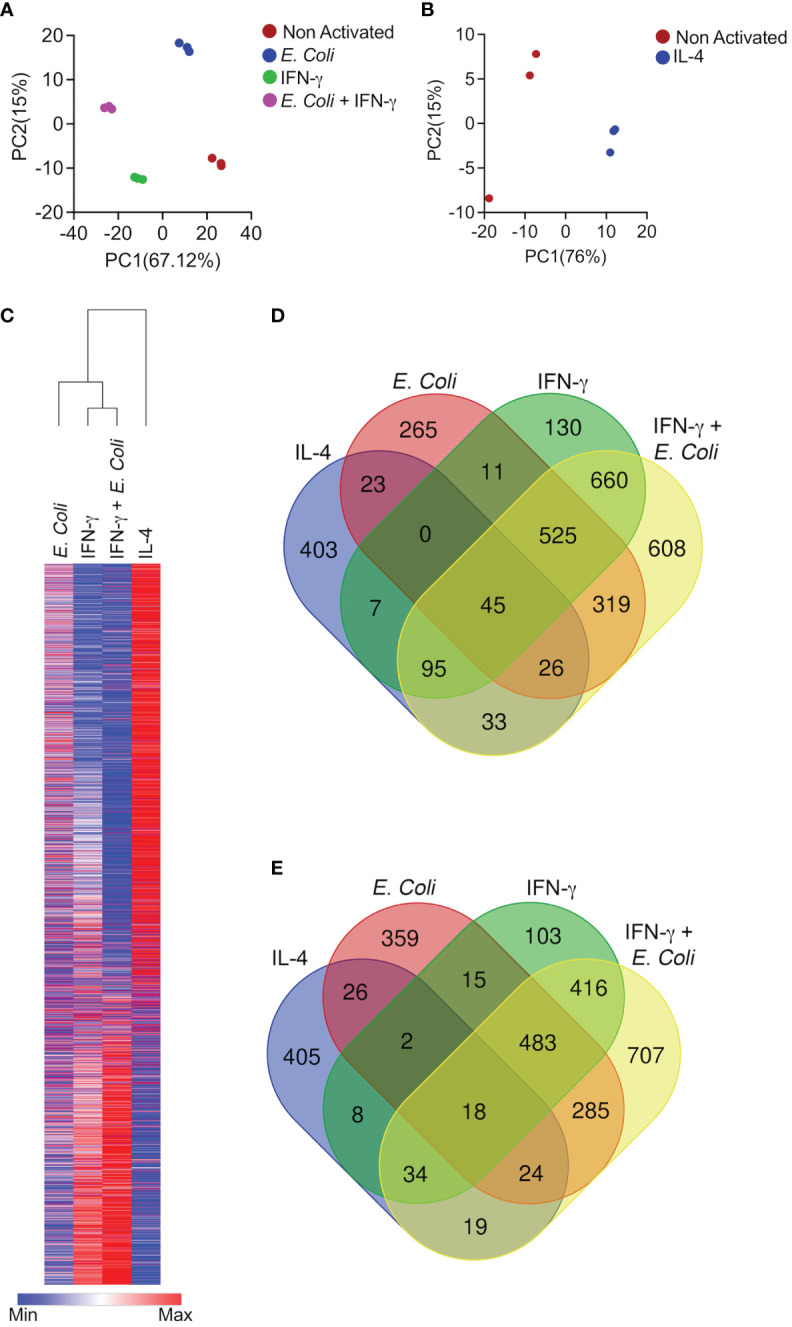
Differential activation of eosinophils results in induction of unique transcriptional programs. Principal components analysis (PCA) of differentially expressed transcripts following activation of eosinophils with *E*. *coli*, IFN-γ, and *E. coli +* IFN-γ **(A)** or IL-4 **(B)**. Heat plot analyses of statistically significant (≥≤ +/-2-fold, adj. p value ≤ 0.05) differentially expressed transcripts from each condition **(C)**,. Venn plot analysis depicting the unique and shared upregulated **(D)** and downregulated **(E)** transcripts following stimulation.

To define whether eosinophils display unique transcriptional signatures following activation with different agents, we chose to stimulate eosinophils with IL-4, IFN-γ, and a combination of innate immune stimulation in the presence of IFN-γ. These settings were specifically chosen since they have been previously shown to represent two extremes of a broad activation spectrum of macrophages, collectively termed “classical” (i.e. M1) and “alternatively activated” (i.e. M2) cells (14). We identified differentially expressed genes based on pairwise comparisons between stimulated and unstimulated eosinophils. Transcripts for which pairwise comparisons between the stimulated and unstimulated groups met statistical significance with adjusted p-value < 0.05, were analyzed. First, we explored the relationships among the biological replicates using principal component analysis (PCA). A PCA plot was generated for unstimulated eosinophils in comparison with eosinophils that were stimulated with IFN-γ, eosinophils stimulated with *E. coli*, and eosinophils that were stimulated with *E. coli* and IFN-γ together (**Figure 1A**). Separation according to the x-axis, which represents principle component 1 (PC1) accounted for approximately 67% of the variance between the stimulated groups. Separation according to the y-axis, which represents PC2 accounted approximately for 15% of the variance between the groups. In addition, a PCA plot was generated for IL-4 stimulated eosinophils (**Figure 1B**). This plot revealed a significant separation between IL-4-stimulated and unstimulated eosinophils with separation according to X-axis-PC1 explaining 76% of the variance.

Comparative analysis of the transcriptome signatures of eosinophils following the diverse stimuli revealed 3,613 genes, which were differentially expressed between the diverse stimuli (**Figure 1C** and **Table S1**).

IL-4 induced a transcriptome signature that consisted of 634 induced transcripts and 536 downregulated transcripts (**Figures 1D, E** and **Table S2**). The majority of these transcripts were unique to stimulation with IL-4 since 63% were uniquely increased (403 transcripts) and 78% (405 transcripts) were uniquely decreased (**Figures 1D, E** and **Table S2**). Activation of eosinophils with IFN-γ alone altered the expression of more than twice as many genes in comparison with IL-4. IFN-γ induced a transcriptome signature that consisted of 1,474 induced transcripts and 1,082 downregulated transcripts (**Figures 1D, E** and **Table S3**). Stimulation of eosinophils with IFN-γ and *E. coli* potentiated the effects of IFN-γ since this combination resulted in the induction of 2,313 transcripts and down regulation of 1,992 transcripts (**Figures 1D, E** and **Table S4**). IFN-γ induced the expression of 130 unique transcripts and decreased the expression of 103 transcripts (**Figures 1D, E** and **Table S5**) whereas the combination of IFN-γ and *E. coli* induced a distinct set of 608 transcripts and decreased the expression of 707 (**Figures 1D, E** and **Table S6**). Interestingly, stimulation of eosinophils with *E. coli* alone induced the distinct expression of 265 transcripts and decreased the expression of 359 (**Figures 1D, E** and **Table S7**).

A major overlap was observed between the transcriptome signature that was induced by stimulations that are “classically” referred to as “Type 1 activation (e.g. IFN-γ and IFN-γ+*E.coli)*. Among the upregulated transcripts, IFN-γ induced 1,326 transcripts, which were also induced by IFN-γ in the presence of *E. coli*. This accounted for 53% of the total transcripts upregulated by IFN-γ and IFN-γ+*E. coli* (**Figure 1D** and **Table S8**). In sharp contrast, IL-4 induced the expression of 199 transcripts, which were induced by IFN-γ in the presence of *E. coli.* (6% of the transcriptome signature). This accounted for 7.2% of the total transcripts upregulated by IL-4 and IFN-γ in the presence of *E. coli* (**Figure 1D**, **Table S9**).

### The Transcriptome Signature of IL-4 Activated Eosinophils

**Figure 2:**
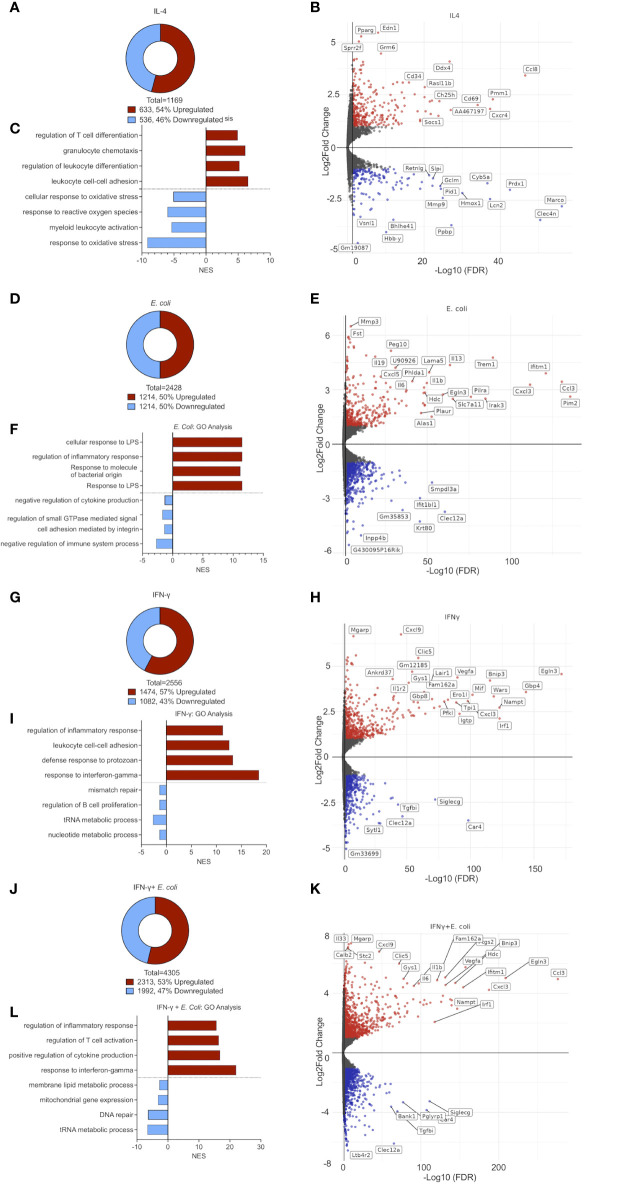
The transcriptome signature of differentially activated eosinophils. Pie chart analysis displaying the number of differentially expressed transcripts (≥≤ +/-2-fold, adj. p value ≤ 0.05) following stimulation of eosinophils with IL-4 **(A)**, *E. coli* **(D)**, IFN-γ **(G)**, and *E. coli +* IFN-γ **(J)**. Volcano-plot representation and partial transcript identities of differentially expressed transcripts following stimulation of eosinophils with IL-4 **(B)**, *E. coli* **(E)**, IFN-γ **(H)**, and *E. coli +* IFN-γ **(K)**. In **(B, E, H, K)** red and blue represent upregulated and downregulated transcripts, respectively. Gene ontology (GO) analysis based on biological processes (BP) using the statistically significant differently express transcripts that were induced by IL-4 **(C)**, *E. coli* **(F)**, IFN-γ **(I)**, and *E. coli+* IFN-γ **(L)**.

Following stimulation of eosinophils with IL-4, we identified 633 upregulated and 536 downregulated transcripts, respectively (**Figure 2A** and **Table S2**). Among the upregulated transcripts, we identified multiple Type 2-related chemokines and chemokine receptors including *Ccl8*, *Ccl17*, *Ccl22*, *Ccl12*, *Ccl24*, *Ccl2* and *Cxcr4* (23) (**Figure 2B**). Moreover, following stimulation with IL-4, eosinophils upregulated the expression of *Cd101* (5.2-fold), and *Cd69* (4-fold) which have been described as markers for recruited (rather than resident) eosinophils (12), and activated eosinophils, respectively (24) (**Figure 2B**). Furthermore, endothelin 1 (*Edn1)*, peroxisome proliferator-activated receptor γ (*Pparg*) and small proline-rich protein 2F (*Sprr2f)*, were markedly upregulated by IL-4 (**Figure 2B**). Conversely, IL-4 downregulated transcripts involved in antimicrobial reaction and inflammation such as interferon-induced transmembrane protein 6 (*Ifitm6*) secretory leukocyte protease inhibitor (*Slpi*), N-formyl peptide receptor 2 (*Fpr2*), (**Figure 2B**).

Gene Ontology (GO)-based on biological processes (BP) analysis revealed that IL-4 provokes cell movement-related pathways. Specifically, “cell chemotaxis”, “leukocyte cell-cell adhesion”, and “regulation of T cell activation” were highly enriched while response of eosinophils to stress such as “response to reactive oxygen species” were markedly decreased (**Figure 2C**).

### The Transcriptome Signature of *E. coli* Activated Eosinophils

Stimulation of eosinophils with *E. coli* resulted in the upregulation of 1,214 transcripts and downregulation of 1,214 transcripts (**Figure 2D** and **Tables S10-11**). This stimulation triggered the expression of various cytokines including pro-inflammatory cytokines (e.g. *Il6*, *Il1b* and *Tnfa*) (25), Type 2 cytokines (e.g. *Il13*) (26, 27) and *Il19*, an LPS-induced immunosuppressive cytokine (28) (**Figure 2E**). Additionally, the expression of several chemokines that are involved in host bacterial infections such as *Cxcl1*, *Cxcl2*, *Cxcl3*, and *Cxcl5* (23) was markedly increased. Furthermore, the expression of multiple proteins that were implicated in innate immune responses and inflammation were increased including triggering receptor expressed on myeloid cells (*Trem1*), paired immunoglobin like type 2 receptor alpha (*Pilra)*, interferon-induced transmembrane protein 1 (*Ifitm1*), interleukin-1 receptor-associated kinase 1 (*Irak1*), *Nfkbiz* (5.2-fold change), *Pou2f2* (7-fold change*)*, *Maff* (13.9-fold change) *Bcl3* (4.1- fold change) and *Arg2* (4.5- fold change) (**Figure 2E**).

GO analysis demonstrated that *E. coli-*activated eosinophils are involved in biological processes such as “response to LPS”, “response to molecule of bacterial origin” “regulation of inflammatory response” and ‘cellular response to LPS’ (**Figure 2F**).

### The Transcriptome Signature of IFN-γ Activated Eosinophils

Following activation with IFN-γ, eosinophils upregulated the expression of 1,474 transcripts and downregulated expression of 1,082 (**Figure 2G** and **Tables S6-7**). IFN-γ activated eosinophils upregulated the expression of multiple cell surface receptors including *Cd274* (programmed death ligand 1) *Ly6a*, *Il13ra1* and leukocyte-associated immunoglobulin-like receptor 1 (*Lair1*), which were previously shown to be regulated by IFN-γ on eosinophils (29, 30). *Cd86*, *Cd53* and *Cd36*, which were described as markers for classically-activated macrophages (M1) (31) were also increased (**Figure 2H**). Among the various secreted factors, which were induced by IFN-γ, CXCL9, a potent chemoattractant for activated T‐cell and NK cells (32), was highly induced (108-fold increase) (**Figure 2H**). Additional hallmark IFN-γ-associated genes were induced such as signal transducer and activator of transcription 1 (*Stat1*), Kruppel-like factor 4 (*Klf4*), interferon regulatory factor-1 and -8 (*Irf1, Irf8*), Interferon-induced transmembrane protein 1 (*Ifitm1*) and Interferon-induced protein with tetratricopeptide repeats 2 (*Ifit2*).

On the other hand, carbonic anhydrase 4 (*Car4)*, transforming growth factor beta-induced (*Tgfbi*) and *Cd48*, which were shown to be markers of eosinophils in setting of allergy and asthma (33), or to support eosinophil adhesion and migration by IL-5 (34), were markedly downregulated (**Figure 2H**).

As expected, the biological processes, which were identified in response to IFN-γ by our GO analysis included “response to IFN-γ”, “defense response to protozoan”, “leukocytes cell-cell adhesion” and “regulation of inflammatory response” (**Figure 2I**).

### The Transcriptome Signature of IFN-γ and *E. coli* Activated Eosinophils

Activation of eosinophils with IFN-γ and *E. coli* resulted in the most robust alteration in transcript levels in comparison with the other treatments, which we examined. Following stimulation, a total of 4,305 transcripts were differentially expressed, where 2,313 were upregulated and 1,992 were downregulated (**Figure 2J** and **Table S4**). The combination of IFN-γ and *E. coli* augmented the expression of various transcripts, which were induced by *E. coli* or IFN-γ alone. For example, *Cd274*, which was increased by 2-fold following stimulation with *E. coli*, and by 6.8-fold by IFN-γ, was further increased to a 10.3-fold increase in the combination of both triggers (**Figure 2K**). Similarly, the expression of several chemokines including *Cxcl1*, *Cxcl2*, *Cxcl3*, *Cxcl5*, and *Cxcl16* as well as the cytokines *Il12b*, *Il27*, *Il33*, and *Il6* were increased to a greater extent following the combination of *E. coli* and IFN-γ in comparison with IFN-γ or *E. coli* alone. Similarly, the expression of *Tgfbi, Car4* and *Siglecg*, were further decreased following the combination of *E. coli* and IFN-γ in comparison with IFN-γ or *E. Coli* alone (**Figure 2K**).

GO analysis revealed that in addition of “regulation of inflammatory response” and “response to IFN-γ“, which were induced by IFN-γ or *E. coli* alone, stimulation of eosinophils with a combination of IFN-γ and *E. coli* enriched biological pathways that regulate cytokine production and T cell activation (**Figure 2L**).

Taken together, these data suggest that eosinophils acquire distinct transcriptome signatures in response to different stimuli, collectively termed Type 1 and Type 2 eosinophils.

### The Unique Transcriptome of Type 1- and Type 2-Activated Eosinophils

**Figure 3:**
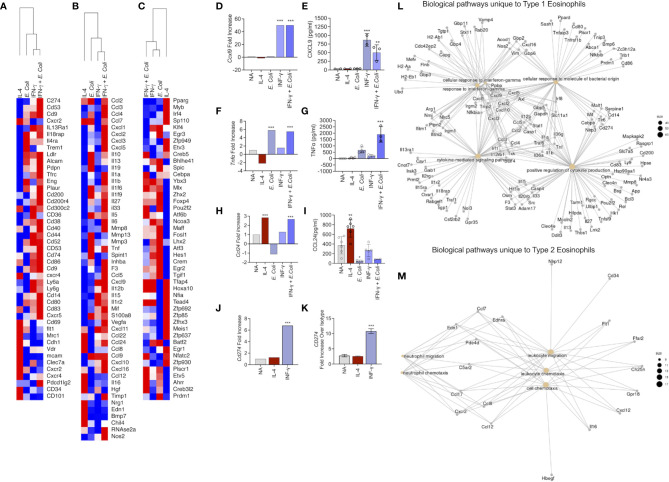
Identification of the unique transcript signatures for Type 1 and Type 2 activated eosinophils Heat plot analysis of differentially expressed cell surface receptors **(A)**, secreted molecules **(B)** and transcription factors **(C)** of eosinophils activated with IL-4, IFN-γ, *E. coli*, and IFN-γ + *E. coli.* The expression of selected transcripts **(D, F, H, J)** was validated in eosinophils by means of ELISA **(E, G, I)** or flow cytometry **(K)**. Gene ontology (GO) analysis based on biological processes (BP) using the statistically significant differently express transcripts that were induced in Type 1 **(L)** and Type 2 **(M)** eosinophils. In **(E, G, I)**, each dot represents a biological replicate, *p < 0.05, **p < 0.01, ***p < 0.001.

Subsequently, we aimed to analyze the different transcriptome profile of eosinophils following exposure to the different stimuli focusing on cell surface markers (**Figure 3A**), soluble mediators (**Figure 3B**) and transcription factors (**Figure 3C**).

#### Cell Surface Receptors

“Type 1” activation increased the expression of multiple co-stimulatory and immunoregulatory receptors such as *Cd74, Cd80, Cd83, Cd86, Cd200, CD274, Cd83*. In addition, several cytokine receptors including *Il4ra, Il13ra1* and *Il18rap* were increased (**Figure 3A**). In contrast, CD101, which was recently described as a marker for inflammatory eosinophils (12), was uniquely upregulated by Type 2 eosinophils (**Figure 3A**). Similarly, CD69, a typical eosinophil activation marker (35), was induced exclusively in Type 2 eosinophils. Moreover, CD34, which was recently shown to play a role in eosinophil migration into allergen-challenged lungs was also uniquely identified in Type 2-activated eosinophils (36).

#### Soluble Mediators

Type 2 activated eosinophils induced the expression of *Ccl22, Ccl24, Ccl8* and *Ccl12*, whereas Type 1 eosinophil activation induced the expression of *Ccl2, Ccl3, Ccl4, Ccl7, Cxcl1, Cxcl2, Cxcl3* and *Cxcl5, Cxcl9 and Cxcl10.* Type 1-activated eosinophils also increased the expression of multiple cytokines including *Il13, Il1a, Il1b, Il33, Il12a, Il12b, Il15* (**Figure 3B**).

#### Transcription Factors

Assessment of transcription factor expression revealed that Type 1 activated eosinophils displayed increased levels of several transcription factors that were associated with M1 macrophages such as *Klf4, Egr3, Atf3, Hes1* and *Crem* (**Figure 3C**). In contrast, Type 2-activated eosinophils increased the expression of various transcription factors that were increased in M2 macrophages (e.g. *Pparg, Irf4*) (**Figure 3C**).

To validate our RNA sequencing analyses, eosinophils were activated and the expression of soluble factors such as CXCL9, TNF-α and CCL24 were determined by ELISA. Consistent with our RNA sequencing data, *Cxcl9* that was induced only by IFN-γ or IFN-γ and *E. coli* (**Figure 3D**), was secreted only by eosinophils that were stimulated with *IFN-γ* or IFN-γ and *E. coli* (**Figure 3E**). Similarly, mRNA expression of *Tnfa* that was increased following activation with IFN-γ or IFN-γ and *E.coli* (**Figure 3F**), was only detected in the supernatants of *E. coli* and to greater extent in supernatants of eosinophils stimulated with IFN-γ and *E.coli* (**Figure 3G**). mRNA expression of *Ccl24*, which was upregulated following activation with IL-4 as well as with IFN-γ and *E.coli* (**Figure 3H**), was significantly increased in the protein level only following stimulation with IL-4 (**Figure 3I**). Finally, *Cd274*, which was increased following IFN-γ and IFN-γ in the presence of *E. Coli* (**Figure 3J**), was also increased by IFN-γ on the surface of eosinophils (**Figure 3K**).

To further dissect the differences between Type 1 and Type 2 eosinophils, we analyzed the unique and shared biological pathways which were enriched in these cells. The unique transcriptome, which was enriched in Type 1 activated eosinophils (**Figure 1D**, 608 transcripts) was associated with “cellular response to IFN-γ”, “cellular response to molecule of bacterial origin”, and “positive regulation of cytokines production” (**Figure 3L**). On the other hand, the unique transcriptome which was enriched in Type 2 activated eosinophils (**Figure 1D**, 403 transcripts) was associated with biological pathways related to cell movement (**Figure 3M**). Importantly, Type 1 and Type 2-activated eosinophils also displayed induction of 33 common transcripts (**Figure 1D**).

### The Shared Transcriptome of Type 1- and Type 2-Activated Eosinophils

**Figure 4:**
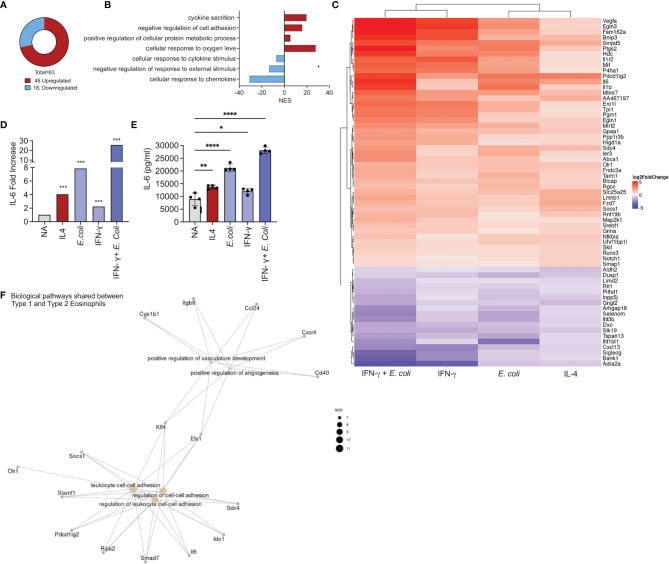
Identification of a common transcript signature for activated eosinophil. Pie chart analysis displaying the number commonly up and downregulated transcripts by all activation conditions [i.e. IL-4, *E. coli*, IFN-γ, and *E. coli +* IFN-γ, **(A)**]. Gene ontology (GO) analysis based on biological processes (BP) using the statistically significant differently expressed transcripts that were commonly up and downregulated in eosinophils following all activation condition **(B)**. Heat plot analysis of the list of transcripts that were commonly up and downregulated by all activation conditions **(C)**. Increased expression of IL-6 transcript levels **(D)** by all activation conditions was validated by activation of eosinophils with IL-4, *E. coli*, IFN-γ, and *E. coli +* IFN-γ and assessment of IL-6 secretion by ELISA **(E)**. Gene ontology (GO) analysis based on biological processes (BP) using the statistically significant mutually expressed transcripts that were induced in Type 1 and Type 2 eosinophils **(F)**. Each dot represents a biological replicate, *p < 0.05, **p < 0.01, ***p < 0.001, ****p < 0.0001.

Our analyses also identified a set of 45 upregulated (**Figure 1C**) and 18 downregulated (**Figure 1D**) transcripts that were shared among all the activation conditions (**Figure 4A**). GO analysis of the biological processes that characterize the commonly regulated transcripts demonstrated that they are associated with biological processes involved in “cytokines secretion, and “negative regulation of cell adhesion” (**Figure 4B**).

Among the common upregulated transcripts various cell surface receptors were identified such programmed cell death 2 (*Pdl2*) and *Il1r2* (**Figure 4C**). In addition, all of the examined stimuli were capable of inducing the expression of *Vegf*, *Il6*, *Il1b* and the transcription factors *Nfikbia* and *Socs1* (**Figure 4C**). Since *Il6* mRNA expression was mutually increased by all of the stimuli (**Figure 4D**), we aimed to determine whether activation of eosinophils with IL-4, *E. Coli*, IFN-γ as well as IFN-γ and *E. coli* will induce IL-6 secretion. Indeed, activation of eosinophils with IL-4, IFN-γ or *E. coli* induced secretion of IL-6. Combination of IFN-γ with *E. coli* augmented the secretion of IL-6 (**Figure 4E**).

Finally, we examined the biological pathways, which were mutually enriched in Type 1 and Type 2 eosinophils. These consist of 33 transcripts (**Figure 1D**) that were associated with angiogenesis and cell adhesion (**Figure 4F**).

### Type 1- and Type 2-Activated Eosinophils Display Distinct Transcriptome Signatures in Comparison With IFN-γ+LPS- and IL-4-Activated Macrophages, Respectively

**Figure 5:**
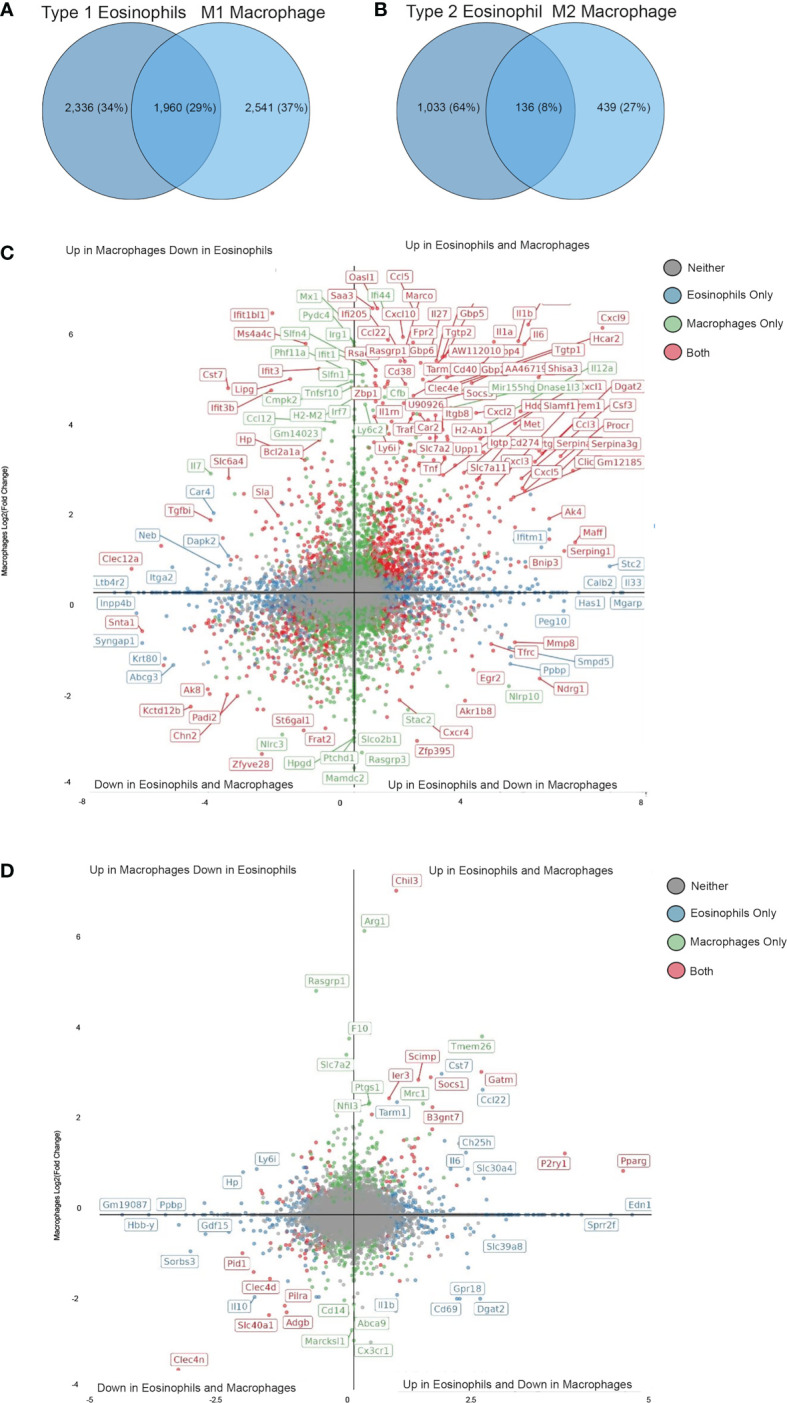
Shared and unique transcriptional profile of Type 1 and Type 2 eosinophils in comparison with M1 and M2 macrophages. Venn plot analysis of statistically significant differentially expressed transcripts from Type 1 **(A)** and Type 2 **(C)** eosinophils in comparison with M1 **(A)** and M2 **(C)** macrophages. Quadrant plot analysis of the statistically significant differentially expressed transcripts from Type 1 **(B)** and Type 2 **(D)** eosinophils in comparison with M1 **(B)** and M2 **(D)** macrophages.

Next, we compared the transcriptome signature of Type 1- and Type 2-activated eosinophils with that of macrophages that have been stimulated with IFN-γ and LPS or IL-4 using a published dataset (37). Type 1-activated eosinophils displayed 1,960 transcripts that were commonly differentially expressed following activation of macrophages with IFN-γ+LPS (**Figure 5A** and **Table S12**). Among these we could identify several “hallmark” inflammatory secreted factors including *Cxcl9. Cxcl10, Tnf, Il6* and *Il1b* (**Figure 5C**) as well as cell surface receptors such as *Tarm1* (T Cell-interacting activating receptor on myeloid Cells 1) and *Cd274*. Notably, among these shared transcripts, several where increased in eosinophils but decreased in macrophages (e.g. *Cxcr4, Mmp8* and *Egr2*, **Figure 5C**) while others where decreased in eosinophils but increased in macrophages (*Tgfbi, Clec12a, Ifit3*). Type 2-activated eosinophils displayed 136 transcripts that were commonly differentially expressed following activation of macrophages with IL-4 (**Figure 5B** and **Table S13**). Among these we identified the previously characterized IL-4-induced transcripts *Chil3*, *Pparg* and *Socs1* (38, 39) (**Figure 5D**). Interestingly, several transcripts that are typically associated with IL-4-activated macrophages were induced only in macrophages (e.g. *Arg1, Mrc1*) (40). In contrast, IL-4 induced the expression of several transcripts specifically in eosinophils such as *Edn1, Sprr2f* (**Figure 5D**). These data demonstrate that while similarities exist between polarized eosinophils and macrophages, each cell type acquires a distinct polarization state.

### Type 1- and Type 2-Activated Eosinophils Resemble Eosinophils From Type 1 and Type 2 Inflammatory Conditions

Since our analyses were conducted using *ex-vivo* stimulations, we were interested to determine whether the transcriptome signature of Type 1- and Type 2-activated eosinophils will resemble the transcriptome of primary eosinophils that were obtained from Type 1 and Type 2 inflammatory conditions. To this end, eosinophils were obtained from the colon of mice undergoing DSS-induced colitis, which is characterized by exposure to microbes and IFN-γ and can thus serve as a Type 1 environment. In addition, eosinophils were sorted from the lungs of mice with experimental asthma (41), which is characterized as a polarized “Type 2” microenvironment with marked elevation of IL-4 (27).

**Figure 6:**
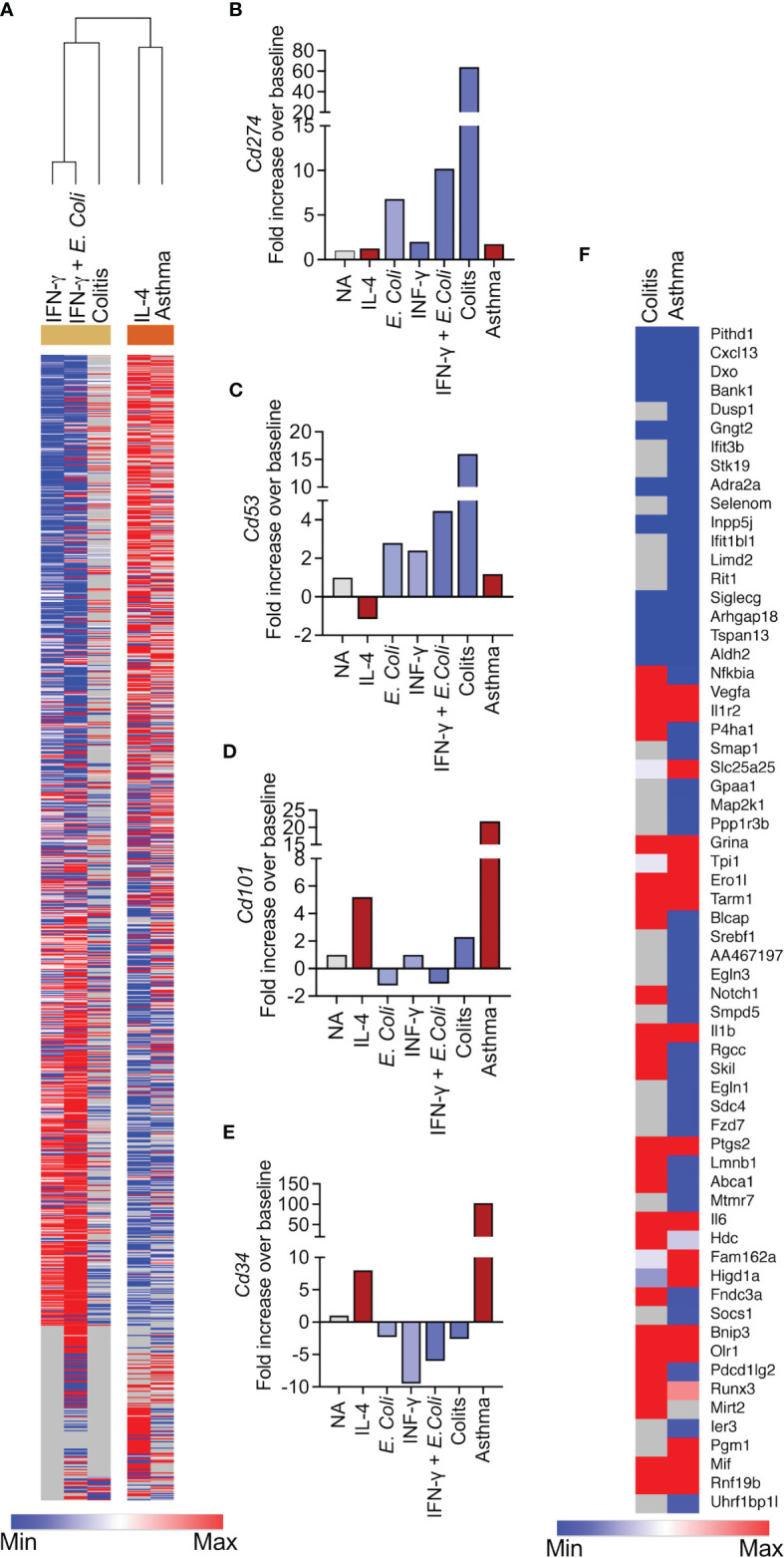
The transcriptional signature of Type 1 and Type 2 eosinophils cluster with the eosinophils from colitis and asthma, respectively. Heatmap analysis of the transcriptional signature of eosinophils activated with IFN-γ*, E. coli+*IFN-γ and IL-4 with the transcriptional signature of eosinophils that were sorted from the lungs of allergen-challenged mice and from the colons of mice undergoing DSS-induced colitis **(A)**. The expression of selected transcripts that were identified as Type 1 **(B, C)** or Type 2 **(D, E)** specific is shown in eosinophils from allergen-challenged mice and mice with colitis **(B–E)**. Heatmap analysis of transcripts that were identified as markers for generally activated-eosinophils in eosinophils obtained from allergen-challenged mice and mice with colitis **(F)**.

Clustering analysis revealed that the transcriptome of Type 1-stimulated eosinophils clustered with eosinophils from the DSS-induces colitis model and Type 2-activated eosinophils clustered with eosinophils from the asthma model (**Figure 6A**, see transcript list in **Table S14**). In support of this, the expression pattern of transcripts that we identified as possible markers for Type 1- and Type 2-activated eosinophils (**Figure 3**), was largely recapitulated in eosinophils from asthmatic mice or mice with colonic inflammation. For example, *Cd274* and *Cd53*, which may serve as markers for Type 1 eosinophils were upregulated in colonic eosinophils following treatment with DSS but not in eosinophils from asthmatic mice (**Figures 6B, C**). In contrast, *Cd101* and *Cd34*, which may serve as markers for Type 2 eosinophils were upregulated in eosinophils from asthmatic mice but not in eosinophils from mice with colonic inflammation (**Figures 6D, E**).

Next, we aimed to validate the shared transcript signature, which we generated from activated eosinophils (**Figure 4**) by comparing it to that of eosinophils from the aforementioned pathophysiological environments. This analysis revealed 12 commonly upregulated (i.e. *Vegfa, Il1r2, Grina, Ero1l, Tarm1, Il1b, Ptgs2, Il6, Bnip3, Olr1*, Mif and Rnf19b) and 10 commonly downregulated transcripts (i.e. *Pithd1, Cxcl13, Dxo, Bank1, Gngt2, Adra2a, Inpp5j, Siglecg, Arhgap18*, Tspan13 and Aldh2) in all of the activation conditions (e.g. IL-4, IFN-γ, *E. coli*, IFN-γ+*E. coli*) and in eosinophils from asthma and colitis (**Figure 6F**).

## Discussion

Eosinophils are key effector cells and therapeutic targets in allergic diseases. Recent data highlight important roles for eosinophils in homeostasis and in inflammatory diseases that are not necessarily associated with allergy (e.g. inflammatory bowel disease and viral infections and even cancer) (2, 9, 42). Thus, upon their recruitment to the inflamed tissue, eosinophils are likely exposed to various stimuli that vary according to the nature of the inflammatory milieu. In fact, similar to other myeloid cells such as macrophages and neutrophils, eosinophils are capable of displaying functional heterogeneity, which is driven by their response to different inflammatory environments. Herein, we characterized the transcriptional landscape of eosinophils in response to IL-4, IFN-γ and *E.* coli, which represent Type 1 and Type 2 inflammatory environments. First, we demonstrated that IL-4, IFN-γ and *E. coli* induced unique transcriptional signatures in eosinophils resulting in enrichment of distinct biological processes. Second, we identified a set of upregulated transcripts, which may serve as Type 1, Type 2 or general activation markers for eosinophils. Finally, we showed that the transcriptional profile of Type 2-activated eosinophils resembled the profile of eosinophils from mice with experimental asthma whereas Type 1 eosinophils resembled eosinophils from mice with colitis. Collectively, these data provide new perspectives regarding eosinophil plasticity and heterogeneity in diverse inflammatory settings and support the notion that eosinophils display a wide continuum of distinct activation states.

Over the past years it has become increasingly apparent that immune cells display marked plasticity (43, 44). Within the myeloid lineage, this has been best characterized in macrophages where *in vitro* modeling of macrophage activations states has been widely applied for molecular and biomarker profiling (14). Consequently, it is now accepted that macrophage activation comprises a wide spectrum of phenotypes which are dictated by the environmental cues that stimulate macrophage activity (14). Similarly, it has been proposed that eosinophils may be classified into different phenotypes as well (9–12). These phenotypes supposedly reflect the potential roles of eosinophils in immune responses and physiological processes. While various eosinophil phenotypes were characterized and the term “E1” and “E2” cells has been suggested, the activation spectrum of eosinophils following Type 1 and Type 2 activation has been largely unexplored. We demonstrate that IL-4 and IFN-γ + *E. coli* stimulated eosinophils are transcriptionally different from each other. We further show that following these stimuli, eosinophils show unique expression of transcripts which can be used to distinguish them as Type 1 and Type 2 eosinophils. Our bioinformatics analysis revealed that Type 2 eosinophils were enriched with pathways that are associated with cell movement. This was evident in the transcriptional profile of Type 2 eosinophils in two ways. First, Type 2 eosinophils upregulated the expression of cell surface molecules that are associated with their movement and tissue infiltration. For example, the surface markers CD101 and CD34 were specifically upregulated in Type 2 eosinophils. CD101 expression was shown to be increased in eosinophils that infiltrate the lungs of allergen-challenged mice and upon infiltration of eosinophils to metastatic lungs (12, 13). Furthermore, *Cd34^-/-^* eosinophils displayed decreased movement *in vitro* and it was shown that CD34 is required for the influx of eosinophils to the gastrointestinal tract and following allergen challenge to the lungs (36, 45). Second, Type 2 eosinophils upregulated and probably secreted multiple chemokines such as *Ccl22, Ccl17, Ccl8* and *Ccl12* that can promote the migration of additional cells in allergic settings including monocytes, T helper cells, basophils and mast cells (46). Importantly, by upregulation and secretion of CCL24, Type 2 eosinophils may also generate a positive feedback loop, which induces the migration of additional eosinophils from the blood. Our data are consistent with previous studies, which assessed the effects of IL-4 and IFN-γ on eosinophils. For example, stimulation of bone marrow-derived mouse eosinophils with IL-4 (or IL-33) for 1 or 4 hours resulted in the induction of 28 transcripts, 9 of which were unique to IL-4 (47). In our hands, 18 hours of stimulation resulted in 633 upregulated transcripts of which 403 were unique to IL-4. Notably, several transcripts were shared in both analyses, including *Ccl2, Ccl7, Cish, Fcrls and lipg*. In addition, while not many studies assessed the effects of IFN-γ on eosinophils, IFN-γ was shown to “prime” eosinophil responses to IL-5 and GM-CSF (48). In addition, several studies have shown that IFN-γ activates eosinophils to secrete chemokines such as CXCL9 and CXCL10 (13, 49). This is consistent with our findings showing that IFN-γ primes eosinophils to innate immune stimulation and is capable of inducing chemokine secretion as well.

Our data provide comprehensive characteristic of subtypes of Type 1-activated eosinophils, which differ from primed eosinophils (IFN-γ activation) or innate immune-activated eosinophils (*E. coli* activation). This classification is supported by our findings of unique transcriptional profiles for each activation set and by the finding that IFN-γ primed eosinophils to innate immune stimulation. Thus, the expression of several transcripts, which were increased by IFN-γ, were further induced in the presence of IFN-γ and innate immune stimulation. These Type 1 eosinophils display a potent pro-inflammatory and immunomodulatory profile that is evident by the robust increase in the number and the identity of their transcripts. Certainly, we identified a set of transcripts that were highly increased in Type 1 eosinophils including expression of innate immune receptors such as CD14, co-stimulatory receptors such as CD80 and CD86 or immune modulating receptors such as CD274 (PD-L1). These data are consistent with previous studies showing the expression of these cell surface markers on eosinophils and especially in activated eosinophils (50). For example, eosinophils were shown to regulate Th1 responses in response to gastric infection *via* IFN-γ-dependent upregulation of CD274 (51). CD274 expression was also increased in colonic tumor-associated eosinophils and metastasis-entrained eosinophils, which were both characterized by an IFN-γ signature (13, 29). Furthermore, the ability of Type 1 eosinophils to upregulate CD80 and CD86, likely enables them to act as antigen presenting cells. In support of this, previous data have shown that eosinophils express and upregulate CD80 and CD86 in response to diverse stimuli including GM-CSF and *Toxocara canis* antigen stimulation (52). Finally, several markers that were previously shown to be expressed by activated eosinophils (53), were also identified in our analyses. These include *Icam1* as well as *Cd9* and *Il2ra.* Of note, Type 1 eosinophils increased the expression of multiple pro inflammatory cytokines including TNF-α, IL-6 and IL-1β as well as chemokines that can induce the recruitment of neutrophils (e.g. CXCL1, CXCL2. CXCL3 and CXCL5). The expression of additional hallmark IFN-γ-induced chemokines such as CXCL9 and CXCL10, which have been shown to induce migration of CD8^+^ T cells (50), was induced as well. Type 1 eosinophils also upregulated various transcripts that are associated with type 2 immunity, such as IL-13, IL-33, IL-13 or IL-4 receptors. Certainly, peripheral LPS injection was shown to markedly increased the surface expression of IL-4 receptor-alpha (IL-4Rα) in microglia. It was suggested that this increase occurs to promote an “anti-inflammatory” M2 phenotype in these cells (54). In addition, LPS-stimulated mast cells were shown to secrete a variety of type 2- and anti-inflammatory-related cytokines including IL-5, IL-10 and IL-13 (55). This suggests that E.coli/IFN-γ induce the upregulation of type 2 related factors, which in turn may generate a negative feedback aiming to limit eosinophil activation by shifting it into a type 2 phenotype

Our study bears the limitation that the eosinophils, which we used were obtained from *Il5^Tg^* mice. Thus, we cannot exclude the possibility that the transcriptional changes that we described are at least partially IL-5-dependent. To partially address this caveat, we compared the transcriptional profile of Type 1 and Type 2 activated eosinophils with the transcriptional profile of eosinophil that were obtained from wild type mice undergoing experimental asthma or colitis. Nonetheless, it is still possible that IL-5 has a role in eosinophil expansion in these models. An additional limitation, which may introduce potential misinterpretation to our data is the fact that our eosinophil population was purified to 95-97% (with contaminating cells were mostly lymphocytes). While this may introduce some caveats, the fact that we were capable of validating our findings by flow cytometry and by ELISA suggests that the highlighted pathways are mostly eosinophil-derived.

Comparing the transcriptional profile of *in vitro* stimulated eosinophils also enabled us to identify potential markers that can be used to detect Type 1 (expressing CD274 and CD53) and Type 2 (expressing CD101 and CD34) eosinophils or even generally activated eosinophils. Indeed, we identified a shared set of transcripts that were commonly upregulated in eosinophils *in vitro* following IL-4 or IFN-γ+*E. coli* stimulation and in primary eosinophils that were obtained from distinct experimental disease models. These include TARM1 (T Cell-Interacting, Activating Receptor On Myeloid Cells 1) (56), which was shown to be expressed on multiple myeloid cells including neutrophils, monocytes, dendritic cells and bone marrow-derived macrophages (56). In addition, IL1R2, a decoy receptor for IL-1 was also upregulated in all the conditions we examined (57). Furthermore, our bioinformatics analyses identified that angiogenesis and endothelial remodeling, which are a feature of asthma (58), were shared biological pathways. Finally, the inflammatory cytokines IL-1β and IL-6 were upregulated by Type 1 and Type 2 eosinophils. Thus, these molecules may serve as general markers for activated eosinophils.

Eosinophils are evolutionary conserved cells, and while several differences exist between mouse and human eosinophils, there are also multiple similarities including their distribution in the body, surface markers and biological functions (59). Emerging evidence support the existence of a spectrum of activation states for human eosinophils as well. For instance, a study on eosinophils in rheumatoid arthritis (RA) suggested the existence of a regulatory eosinophils (based on the expression of CD62L) in patients with remission (8). Similar results were found in lung eosinophils from healthy and asthmatic patients suggesting that inflammatory eosinophils were negative for CD62L expression, whereas resident regulatory eosinophils expressed variable levels of CD62L (12). In our data CD62L (termed *Sell* in our datasets) was significantly down regulated by *E. co*li and IFNγ+ *E. coli* (-1.72 and-1.69 respectively). This possibly indicates that these eosinophils lose their regulatory capacity.

In conclusion, using an unbiased global profiling approach followed by bioinformatics analyses, we characterized the transcriptional plasticity of eosinophils. Our findings provide important insights into the regulation of eosinophil gene expression and provide potential biomarkers for their activation state. We provide fundamental knowledge regarding the heterogeneity of eosinophils and support the presence of transcriptional differences between Type 1 and Type 2 cells that is likely reflected by their pleotropic activities in diverse disease settings.

## Data Availability Statement

The data presented in the study are deposited in the NCBI GEO repository, accession number GSE189213.

## Ethics Statement

The animal study was reviewed and approved by Animal Health Care Committee of the Tel Aviv University.

## Author Contributions

AD and AM - conception and/or design of the work. AD, SG-T, IH, SA, YG, and MI- Data Collection. AD, GS, NS, and AM- Data analysis and interpretation. AD and AM- Drafting the article. GS, SG-T, IH, SA, YG, MI, and NS - Critical revision of the article. All authors contributed to the article and approved the submitted version.

## Funding

NS is supported by the Israel Science Foundation (ISF; 1852/16); Horizon 2020 - Research and Innovation Framework Programme, PSY-PGx; Israeli Ministry of Defense, Office of Assistant Minister of Defense for Chemical, Biological, Radiological and Nuclear (CBRN) Defense; The Edmond J. Safra Center for Bioinformatics at Tel Aviv University; The Koret-UC Berkeley-Tel Aviv University Initiative in Computational Biology and Bioinformatics; The QBI/UCSF-Tel Aviv University joint Initiative in Computational Biology and Drug Discovery; Tel Aviv University Richard Eimert Research Fund on Solid Tumors; Collaborative clinical Bioinformatics research of the Edmond J. Safra Center for Bioinformatics and Faculty of Medicine at Tel Aviv University; Israeli Ministry of Science and Technology, Israeli–Russia; The Center for Combating Pandemics at Tel Aviv University; and a generous donation from the Adelis Foundation. AM is supported by the US-Israel Bi-national Science Foundation (grant no. 2015163), by the Israel Science Foundation (grants no. 886/15 and 542/20), the Israel Cancer Research Fund, the Richard Eimert Research Fund on Solid Tumors (TAU), the Israel Cancer Association Avraham Rotstein Donation, the Cancer Biology Research Center (TAU) and the Emerson Collective. GS is a PhD fellow of the Edmond J Safra Center for Bioinformatics at Tel Aviv University and is partially supported by the center.

## Conflict of Interest

The authors declare that the research was conducted in the absence of any commercial or financial relationships that could be construed as a potential conflict of interest.

## Publisher’s Note

All claims expressed in this article are solely those of the authors and do not necessarily represent those of their affiliated organizations, or those of the publisher, the editors and the reviewers. Any product that may be evaluated in this article, or claim that may be made by its manufacturer, is not guaranteed or endorsed by the publisher.
